# Dynamic computed tomography findings of an accessory spleen in the pelvis: a case report

**DOI:** 10.1186/s40792-016-0152-7

**Published:** 2016-03-13

**Authors:** Hiroshi Ota, Yasutomo Ojima, Daisuke Sumitani, Masazumi Okajima

**Affiliations:** Department of Surgery, Hiroshima City Hiroshima Citizens Hospital, 7-33, Motomachi, Naka-ku, Hiroshima City, 730-5818 Japan; Department of Gastroenterological and Transplant Surgery, Applied Life Sciences, Institute of Biomedical and Health Sciences, Hiroshima University, 1-2-3, Kasumi, Minami-ku, Hiroshima City, 734-8551 Japan

**Keywords:** Accessory spleen, Pelvis, Vascular pedicle, Dynamic computed tomography

## Abstract

We report the case of a 60-year-old man with an accessory spleen in the pelvis. He visited our outpatient clinic because of abdominal discomfort. Computed tomography (CT) showed an enhanced mass (40 mm in diameter) in the pelvis. Preoperative diagnosis was difficult even after magnetic resonance imaging and colonoscopy. The patient underwent surgery for suspicion of a gastrointestinal stromal tumor or malignant lymphoma of the rectum. Intraoperative findings showed a mass in the pelvis and a long cord-like tissue reaching the mass and arising from the great omentum; the mass was excised. Histopathologic examination indicated that the mass was splenic tissue, and feeding vessels were found in the cord-like tissue, which were determined to be derived from the left gastroepiploic artery and vein. Thus, we diagnosed it as an accessory spleen in the pelvis. An accessory spleen is not rare and can occur anywhere in the abdominal cavity. However, an accessory spleen in the pelvis is an infrequent finding, and only 9 other cases of an accessory spleen in the pelvis have been reported. Therefore, it is very difficult to make a correct diagnosis preoperatively. However, 7 of the 9 cases (77.8 %) of a pelvic accessory spleen had vascular pedicles from the great omentum or splenic hilum as feeding vessels; hence, determining the feeding blood vessels on dynamic CT may be useful for diagnosing an accessory spleen in the pelvis. Additionally, if the accessory spleen is symptomatic or has a vascular pedicle, surgeons should attempt to resect the accessory spleen in the pelvis using minimally invasive laparoscopy.

## Background

Accessory spleens are not rare and are commonly found in the splenic hilum, great omentum, and pancreas [1]. However, an accessory spleen in the pelvis is a very rare finding. As most accessory spleens in the pelvis are suspected to be ovarian tumors depending on their location [2–5], it is very difficult to make a correct diagnosis preoperatively. We report the case of an accessory spleen in the pelvis that was challenging to diagnose preoperatively.

## Case presentation

**Fig. 1:**
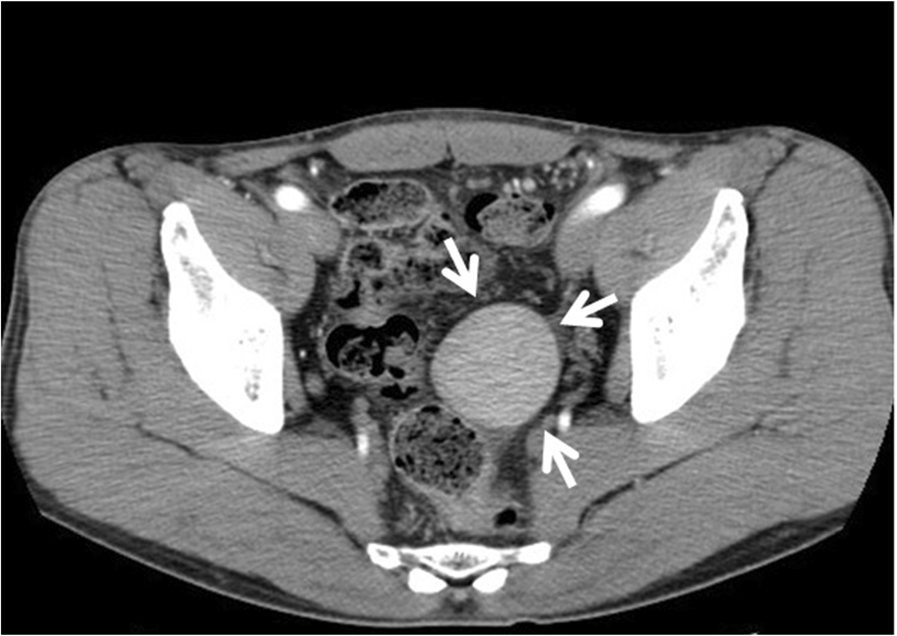
The pelvic mass on dynamic computed tomography. A 40-mm solid pelvic mass was observed anterior to the rectum and was enhanced homogeneously (*arrow*)

**Fig. 2:**
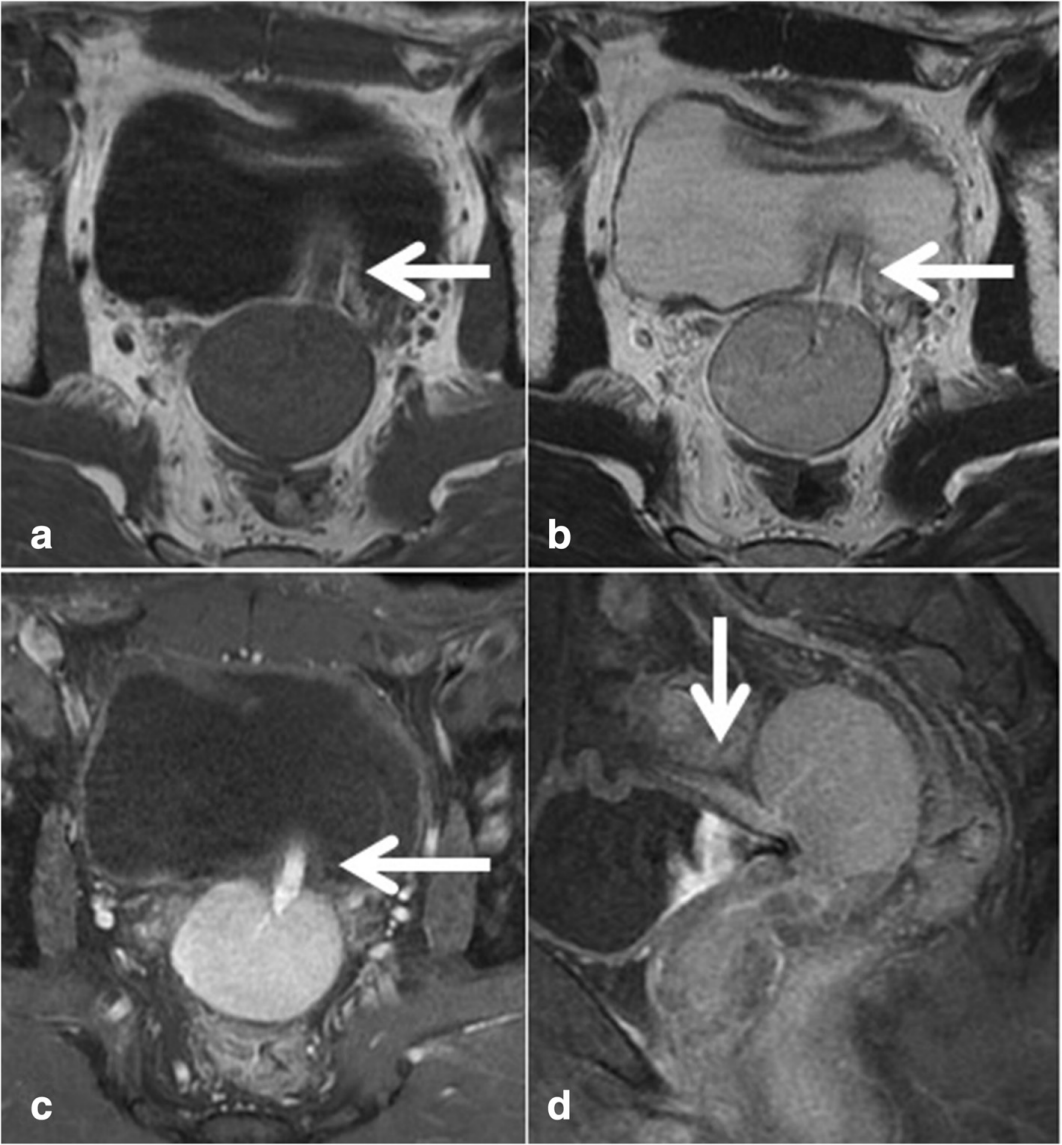
Magnetic resonance imaging (MRI) of the pelvic mass. The pelvic mass measured 46 × 47 × 38 mm and exhibited a low signal on T1-weighted imaging (**a**), a slightly high signal on T2-weighted imaging (**b**), and a high signal on contrast-enhanced imaging (**c**). MRI also showed that a blood vessel extended to the ventral side of the mass (**d**, *arrow*; coronal image)

A 60-year-old man visited our outpatient clinic because of lower abdominal discomfort in the 2 months preceding his visit. He had a history of chronic hepatitis C infection and cirrhosis. There was no history of abdominal trauma. The abdomen was soft, and no mass was palpable. The hemoglobin concentration, white blood cell count, platelet count, and C-reactive protein level were within normal limits. The tumor marker levels were also within normal ranges, such as CEA 2.9 ng/mL and CA19-9 25 U/mL. Dynamic computed tomography (CT) showed a 40-mm solid pelvic mass anterior to the rectum. The mass was enhanced homogeneously with no infiltration into the organs surrounding the mass (Fig. 1). The spleen was visualized in the left upper quadrant, and mild splenomegaly was present. Magnetic resonance imaging (MRI) showed that the pelvic mass measured 46 × 47 × 38 mm, and it had a low signal on T1-weighted imaging and a slightly high signal on T2-weighted imaging. MRI also showed a blood vessel that extended to the ventral side of the mass (Fig. 2a–d). Since the MRI was confined to the pelvis, the origin of the vascular pedicle could not be identified. We suspected that the feeding blood vessels were derived from the mesenteric vessels. Abnormal accumulation was not detected on positron emission tomography (PET)-CT. No abnormalities were found in the mucous membrane of the rectum, and no clear compressive lesion was detected on colonoscopy. Based on these findings, a preoperative diagnosis was difficult to make. Since the mass was near the rectum and had blood flow from the mesenteric vessels, we preoperatively suspected a gastrointestinal stromal tumor (GIST) or malignant lymphoma in the rectum.

**Fig. 3:**
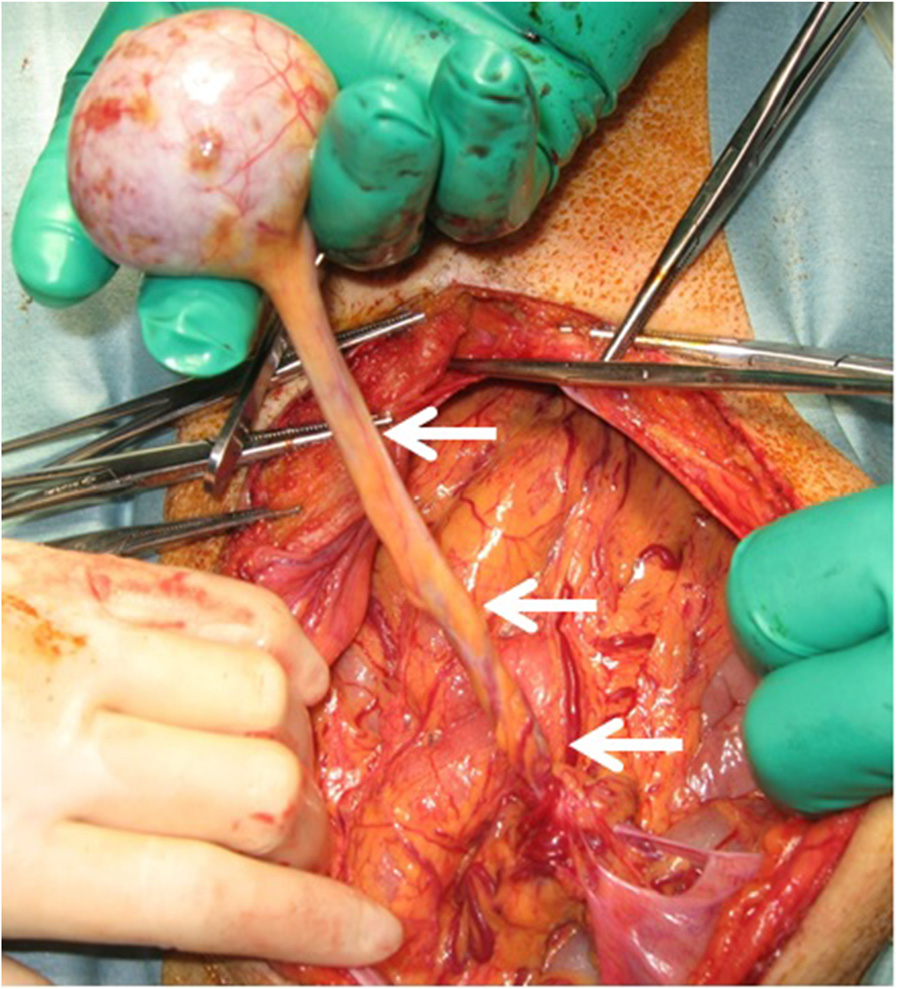
Intraoperative findings. A spherical firm mass was found in the pelvis. The mass had a long, cord-like tissue arising from the great omentum (*arrows*)

**Fig. 4:**
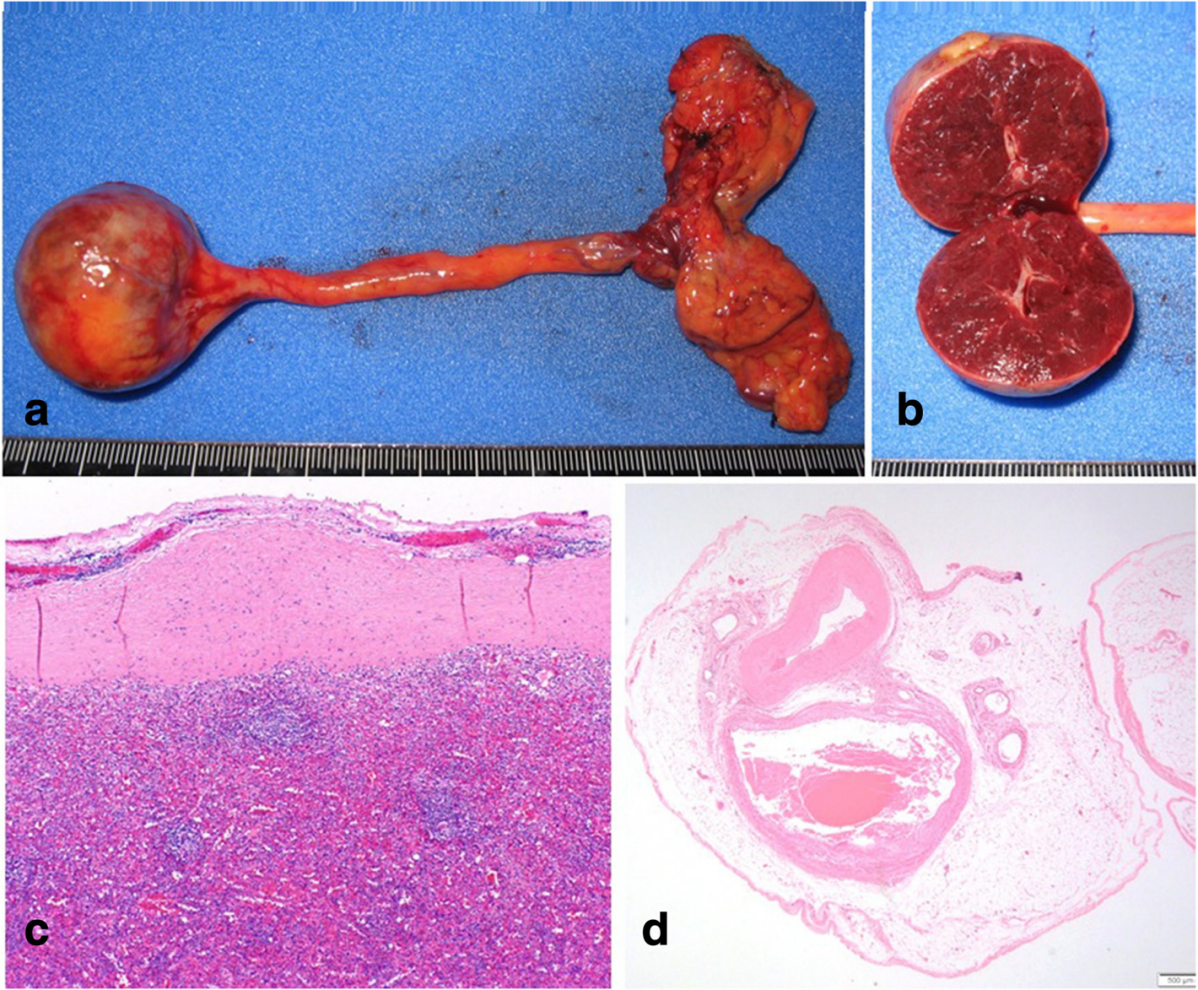
Histological findings. The mass measured 45 × 37 mm **(a)** and the cut surface was red and solid **(b)**. Histopathologic examination showed that the resected mass was splenic tissue consisting of red pulp and white pulp **(c)**, and feeding vessels were found in the cord-like tissue (**d**, *arrow*)

Intraoperative findings showed a spherical firm mass with a well-defined capsule, measuring approximately 4 cm in diameter. The mass had a long cord-like tissue arising from the great omentum (Fig. 3) and was surgically removed after ligation of the cord-like tissue. Histopathologic examination showed that the resected mass was splenic tissue consisting red pulp and white pulp, and feeding vessels were found in the cord-like tissue (Fig. 4a–d). The feeding vessels were determined to be derived from the left gastroepiploic artery and vein and not the mesenteric vessels. As the spleen was visualized in the left upper quadrant, the mass was diagnosed as an accessory spleen in the pelvis. The patient was discharged on the ninth postoperative day, and follow-up examination 4 years later showed no abnormal findings.

### Discussion

Dogan et al. [1] reported that accessory spleens were found in 48 (6.7 %) of 720 autopsy cases, which indicated that it is not a rare occurrence. They also reported that accessory spleens were found in the hilum of the main spleen in 28 cases (58.3 %), great omentum in 13 (27.0 %), and pancreas in 5 cases (10.4 %). Of the 720 cases, only 2 had accessory spleens in the pelvis; thus, the pelvis was classified as an atypical localization for an accessory spleen.

**Fig. 5:**
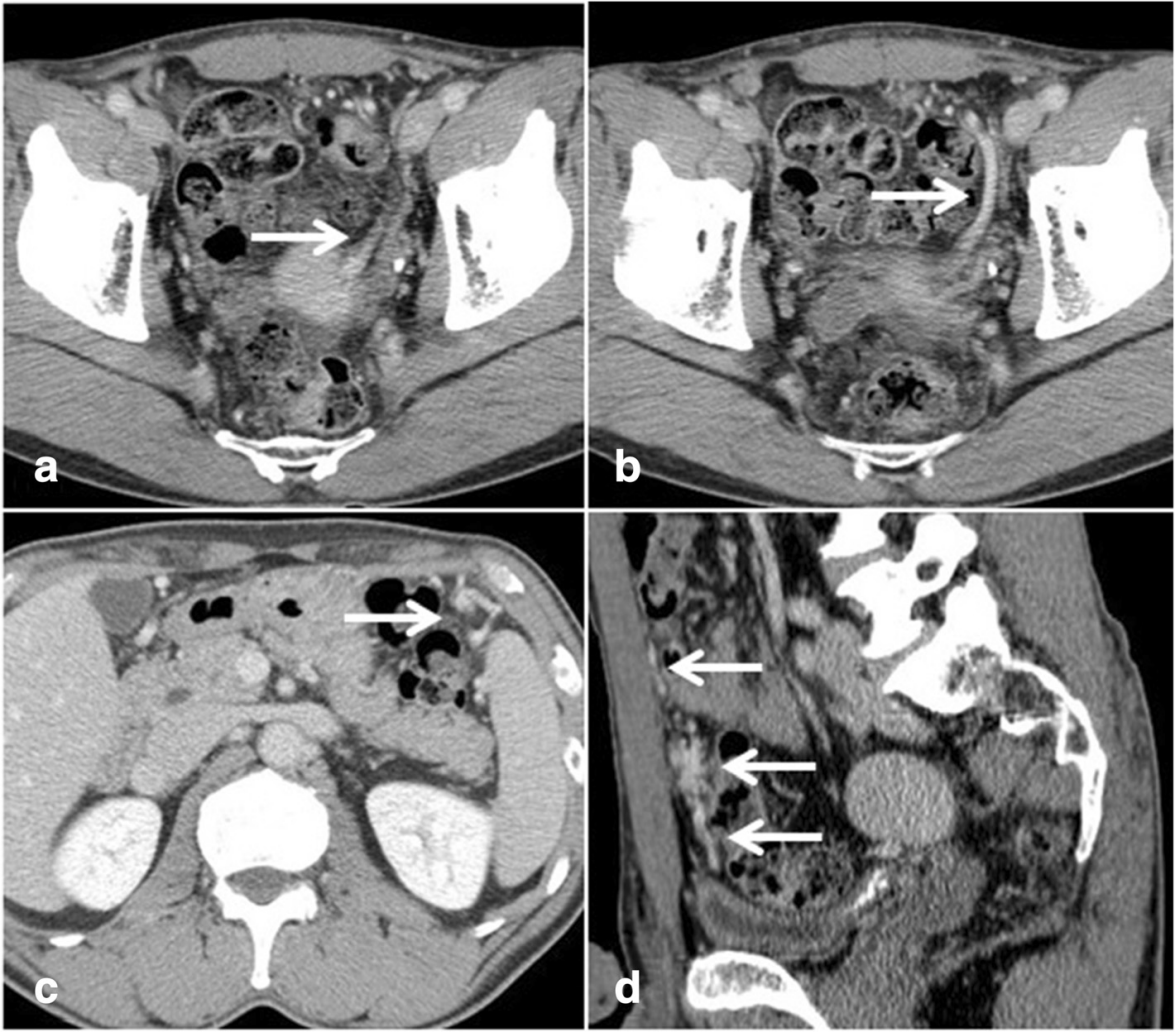
Preoperative computed tomography. Dynamic computed tomography shows the feeding blood vessel of the mass (**a**, *arrow*), which extended to the ventral side (**b**, *arrow*), and was derived from the left gastroepiploic artery (**c**, *arrow*). Coronal image shows that the vessel ascended toward the ventral side (**d**, *arrows*; coronal image)

A PubMed search of the literature using the keywords “accessory spleen” and “pelvis” resulted in the identification of only 9 cases of an accessory spleen in the pelvis [2–9], including our case (Table 1). These cases included 3 men and 6 women, ranging in age from 17 to 75 years (median, 43 years). Although accessory spleens are mostly asymptomatic, 8 patients (88.9 %) with a pelvic accessory spleen were reported to exhibit symptoms such as abdominal pain (5 patients), abdominal discomfort (2 patients), and anemia (1 patient). The median maximum mass diameter was 5.0 cm (range 4.0–13.0 cm). A pelvic accessory spleen is difficult to suspect preoperatively; only 2 of the 8 patients were diagnosed with an accessory spleen in the pelvis preoperatively [7, 8]. Due to the occurrence of many of these cases in women, 4 were suspected to be ovarian tumors and 1 was suspected to be a recurrence of a renal tumor and GIST in the rectum. These aforementioned cases were diagnosed as an accessory spleen based on laparotomy or histopathological findings, underscoring the difficulty of diagnosing this disease preoperatively. Nishiguchi et al. [7] reported that imaging methods such as ultrasonography, CT, and MRI do not show signals specific to an accessory spleen. They also reported that scintigraphy with Tc-99m phytate is the most useful in evaluating an accessory spleen [7]. However, scintigraphy is difficult to be performed immediately after the incidental detection of a pelvic mass. In addition, we did not consider scintigraphy in our case even after several examinations were performed. Although MRI confirmed the thick feeding blood vessel connected to the mass, we thought it was a blood vessel from the mesentery. Postoperative reassessment of the preoperative dynamic CT findings showed that the vessel was derived from the gastroepiploic artery (Fig. 5a–d). Preoperative confirmation of this finding along with scintigraphy would have enabled us to diagnose the mass as an accessory spleen.

**Table 1:** Summary of reported cases of accessory spleen in the pelvis

Number	Author	Year	Age	Sex	Symptom	Tumor size (cm)	Preoperative diagnosis	Operation	Vascular pedicle	Origin of feeding vessels
1	Wood [6]	1987	38	M	No	6	Unknown	Tumor resection	Yes	Splenic hilum
2	Azar [2]	1993	44	F	Left lower abdominal pain	7	Left ovarian tumor	Tumor resection	Yes	Splenic hilum
3	Vural [3]	1999	26	F	Left lower abdominal pain	4.5	Subserous myoma, ectopic spleen, ovarian tumor	Tumor resection	Yes	Gastroepiploic artery
4	Nishiguchi [7]	2001	58	F	Lower abdominal discomfort	4	Accessory spleen	No	Yes	Splenic hilum
5	Hisao [4]	2001	17	F	Lower abdominal pain	13	Right ovarian tumor	Tumor resection	Yes	Gastroepiploic artery
6	Cowles [8]	2007	18	M	Lower abdominal pain	5	Accessory spleen	Laparoscopic tumor resection	Yes	Splenic hilum
7	Ruiz-Tovar [9]	2009	75	F	Anemia	5	Recurrence of a renal tumor	Tumor resection	Unknown	Unknown
8	Taskin [5]	2015	43	F	Left lower abdominal pain	5.5	Ovarian tumor	Tumor resection	No	No
9	Our case	2015	60	M	Abdominal discomfort	4.7	GIST in the rectum	Tumor resection	Yes	Gastroepiploic artery

Additionally, we found that in 7 of the 9 cases (77.8 %) in the previous studies, the feeding vessels originated from the vascular pedicles from the great omentum or splenic hilum [2–4, 6–8]. Thus, determination of the origin of the feeding blood vessels either from the omental artery or splenic hilum on pre-operative dynamic CT may be useful for the differential diagnosis of an accessory spleen in the pelvis. However, Suzuki et al. [10] and Todoroki et al. [11] also reported the presence of feeding arteries originating from the gastroepiploic artery of an omental GIST; hence, it is important to differentiate between an accessory spleen in the pelvis and omental GIST. Scintigraphy with Tc-99m phytate may be useful in the case of a differential diagnosis of either an accessory spleen or omental GIST. Additionally, GIST often shows abnormal accumulation upon PET-CT, and abnormal accumulation was not detected in our case. Hence, we consider PET-CT as one of the suitable options.

Surgery is recommended in cases of a pelvic accessory spleen with a vascular pedicle, because the pedicle may cause abdominal symptoms due to torsion, as reported by Vural et al. [3] that torsion of the pedicle leads to rupture and infarction. Morita et al. [12] described the following characteristics associated with torsion of an accessory spleen: (1) a mass 40 mm or more in diameter, (2) an ectopic presentation (e.g., in the tail of the pancreas or omentum), (3) women aged 40 years or younger, (4) the presence of abdominal pain, (5) the presence of a feeding blood vessel, and (6) the lack of supporting tissue. Surgery for tumor resection was performed in all 8 cases. Open surgery was performed in 7 of the 8 cases, but Cowles et al. [8] reported laparoscopic tumor resection. Since our case met the aforementioned first, second, fifth, and sixth characteristics, surgery was appropriate. Moreover, in our case, even if the mass was suspected to be a pelvic accessory spleen preoperatively, laparoscopic surgery would have been possible to resect it. An accessory spleen in the pelvis with a vascular pedicle appears to be a good indication for minimally invasive laparoscopic surgery. Thus, surgeons should consider laparoscopic resection of a pelvic mass with a vascular pedicle.

## Conclusions

Surgeons should confirm the presence or absence of a vascular pedicle on dynamic CT upon incidental identification of a pelvic mass. Additionally, laparoscopic surgery should be considered for the resection of an accessory spleen in the pelvis with a vascular pedicle.

## Consent

Written informed consent was obtained from the patient for publication of this case report and any accompanying images. A copy of the written consent is available for review by the Editor-in-Chief of this journal.

## Abbreviations

CT: computed tomography
GIST: gastrointestinal stromal tumor
MRI: magnetic resonance imaging
PET-CT: positron emission tomography-computed tomography
